# *Candida lusitaniae* in Kuwait: Prevalence, antifungal susceptibility and role in neonatal fungemia

**DOI:** 10.1371/journal.pone.0213532

**Published:** 2019-03-07

**Authors:** Ziauddin Khan, Suhail Ahmad, Noura Al-Sweih, Seema Khan, Leena Joseph

**Affiliations:** 1 Department of Microbiology, Faculty of Medicine, Kuwait University, Safat, Kuwait; 2 Microbiology Department, Maternity Hospital, Shuwaikh, Kuwait; Center for Cancer Research, UNITED STATES

## Abstract

**Objectives:**

*Candida lusitaniae* is an opportunistic yeast pathogen in certain high-risk patient populations/cohorts. The species exhibits an unusual antifungal susceptibility profile with tendency to acquire rapid resistance. Here, we describe prevalence of *C*. *lusitaniae* in clinical specimens in Kuwait, its antifungal susceptibility profile and role in neonatal fungemia.

**Methods:**

Clinical *C*. *lusitaniae* isolates recovered from diverse specimens during 2011 to 2017 were retrospectively analyzed. All isolates were identified by germ tube test, growth on CHROMagar Candida and by Vitek 2 yeast identification system. A simple species-specific PCR assay was developed and results were confirmed by PCR-sequencing of ITS region of rDNA. Antifungal susceptibility was determined by Etest. Minimum inhibitory concentrations (MICs) were recorded after 24 h incubation at 35°C.

**Results:**

Of 7068 yeast isolates, 134 (1.89%) were identified as *C*. *lusitaniae* including 25 (2.52%) among 990 bloodstream isolates. Species-specific PCR and PCR-sequencing of rDNA confirmed identification. Of 11 cases of neonatal candidemia, 9 occurred in NICU of Hospital A and are described here. Eight of 9 neonates received liposomal amphotericin B, which was followed by fluconazole in 7 and additionally by caspofungin in 2 cases as salvage therapy. Three of 8 (37.5%) patients died. No isolate exhibited reduced susceptibility to amphotericin B, fluconazole, voriconazole, caspopfungin, micafungin and anidulafungin. The MIC ± geometric mean values for amphotericin B, fluconazole, voriconazole, and caspofungin were as follows: 0.072 ± 0.037 μg/ml, 2.32 ± 0.49 μg/ml, 0.09 ± 0.01 μg/ml and 0.16 ± 0.08 **μg**/ml, respectively. Only two isolates exhibited reduced susceptibility to fluconazole.

**Conclusions:**

This study describes the prevalence and antifungal susceptibility profile of clinical *C*. *lusitaniae* isolates in Kuwait. No isolate showed reduced susceptibility to amphotericin B. The study highlights the emerging role of *C*. *lusitaniae* as a healthcare-associated pathogen capable of causing fungemia in preterm neonates and causing significant mortality.

## Introduction

*Candida lusitaniae* (teleomorph *Clavispora lusitaniae*) was first described by van Uden and Carmo-Sousa as a common flora in the gastrointestinal tract of warm blooded animals [1]. It was recognized as a human pathogen in three patients with septicemia and in two of them, the isolates were reported as variants of *C*. *tropicalis* [2, 3]. The first documented case of opportunistic infection where *C*. *lusitaniae* strain developed resistance to amphotericin B during therapy was published in 1979 [4,5]. Over the years, there has been a gradual increase in the number of cases with *C*. *lusitaniae* infection, predominantly in cancer patients who received bone marrow transplantation or cytotoxic chemotherapy [6,7]. Some strains are known to exhibit intrinsic or acquired resistance to amphotericin B [5, 8–11]. This species is being increasingly isolated from cancer patients on empirical/prophylaxis antifungal therapy [12, 13]. A study from Anderson Cancer Center (Houston, Texas) during 2006–2013 revealed a high rate of occurrence of *C*. *lusitaniae* among cancer patients (1.45 episodes/100,000 inpatient days) and it was also the third most common cause of breakthrough candidemia (7/37, 19%) associated with 53% mortality [13]. In Kuwait, little information is available on the association of *C*. *lusitaniae* with human colonization and infection [14]. This study describes prevalence and susceptibility profile of clinical isolates of *C*. *lusitaniae* and its role in neonatal fungemia in Kuwait.

## Materials and methods

### Reference strains, clinical isolates and phenotypic identification

Reference strains or well characterized clinical isolates of *C*. *lusitaniae* (CBS4413, CBS1944, CBS6936 and ATCC38533), *Candida dubliniensis* (CD36), *Candida albicans* (ATCC56881), *Candida africana* (CBS9118), *Candida parapsilosis* (ATCC22019), *Candida orthopsilosis* (ATCC96139), *Candida metapsilosis* (ATCC96143), *Candida glabrata* (ATCC90030), *Candida krusei* (ATCC6258), *Candida tropicalis* (ATCC34139), *Meyerozyma guilliermondii* (CBS6021), *Candida kefyr* (ATCC28838), *Candida conglobata* (Kw381/16), *Candida utilis* (Kw3642/15), *Candida haemulonii* (Kw154/06), *Candida duobushaemulonii* (Kw3270/08) and *Candida auris* (Kw2611/17) were used as reference *Candida* species. All clinical *C*. *lusitaniae* isolates included in the study were obtained between January 2011 and December 2017. They were isolated from different clinical specimens in microbiology laboratories of various hospitals across Kuwait and were referred to Mycology Reference Laboratory (MRL) for identification and antifungal susceptibility testing as part of routine patient care. The clinical specimens were collected after obtaining informed verbal consent and this procedure was approved by the ethical Committee, Ministry of Health, Kuwait and Health Sciences Center, Kuwait University. The specimens were cultured on Sabouraud dextrose agar supplemented with chloramphenicol and growth was streaked to get isolated colonies to ensure purity of culture. All blood culture isolates were obtained using BACTEC 9240 system or BACTEC Peds Plus/F culture bottles (Becton Dickinson, Paramus, NJ, USA). All positive cultures were Gram-stained and subcultures were made on blood agar and/or Sabouraud dextrose agar. Isolated yeast colonies were processed for routine identification procedures, which included direct microscopic examination by wet mount, Germ tube test, growth characteristics on CHROMagar Candida and identification by Vitek 2 yeast identification system [15]. Laboratory details of all the isolates identified as *C*. *lusitaniae* since 2011 were retrospectively collected for source of isolation. All bloodstream isolates were also subjected to identification by matrix assisted laser desorption ionization-time of flight mass spectrometry (MALDI-TOF MS), performed as described previously [16].

### Molecular characterization

Genomic DNA was extracted from reference strains or clinical isolates from 1 ml of cell suspension in Sabouraud dextrose broth by Gentra Puregene Yeast DNA extraction kit (Qiagen, Hilden, Germany) according to kit instructions or by the rapid method using Chelex-100 as described previously [17]. A simple, low-cost PCR assay was developed for rapid molecular identification of *C*. *lusitaniae* isolates. For this purpose, one forward (CLUSITF, 5’-TTGYWTTTGCGAACAAAAAAA-3’) and one reverse (CLUSITR, 5’-TATTTCGGAGCAACGCCTA-3’) primer targeting specific sequences within ITS-1 and ITS-2 regions of rDNA of *C*. *lusitaniae* were synthesized. The unique primer sequences designed in this study were based on sequence alignment of ITS region sequences from multiple strains of all commonly encountered clinical yeast species that are available from the GenBank. The species specificity of the primers CLUSITF and CLUSITR for *C*. *lusitaniae* was indicated by BLAST searches (http://www.ncbi.nlm.nih.gov/entrez/query.fcgi) as they showed complete sequence identity only with *C*. *lusitaniae* strains. The reaction and PCR cycling conditions were same as described previously except that primers CLUSITF and CLUSITR were used [17]. PCR amplicons were run on 2% (w/v) agarose gels, as described previously [18]. The results of species-specific identification of all *C*. *lusitaniae* isolates were confirmed by DNA sequencing of the ITS region of rDNA. The ITS region was amplified by using panfungal primers (ITS1 and ITS4) and both strands were sequenced as described previously [19, 20]. BLAST searches (http://blast.ncbi.nlm.nih.gov/Blast.cgi?) were performed and >99% sequence identity with corresponding sequence from *C*. *lusitaniae* reference strains (CBS6936 or CBS5094 or CBS4413 or ATCC38533) available in the GenBank was used for species identification [21].

Fingerprinting of *Candida* species isolates by molecular techniques is performed for epidemiological studies [22]. The genotypic relationship among bloodstream *C*. *lusitaniae* isolates collected from neonates in Hospital A was studied by comparing ITS region of rDNA sequences. The ITS region of rDNA sequences from 2 other bloodstream isolates; *C*. *lusitaniae* Kw718/11 and *C*. *lusitaniae* Kw1058/15 collected from 2 neonates from Hospital B during the period of this study and a bloodstream isolate (*C*. *lusitaniae* Kw2009/07) collected 2 years earlier from a neonate from Hospital A were also used. The rDNA sequences from *C*. *lusitaniae* CBS6936, *C*. *lusitaniae* CBS5094, *C*. *lusitaniae* CBS4413 and *C*. *lusitaniae* ATCC38533 available from GenBank were also retrieved and used for comparisons. Multiple sequence alignments were performed with Clustal omega and the phylogenetic tree was constructed with MEGA 6.1 software by using the Neighbor-joining method with Kimura-2 parameter model, as described previously [23]. The robustness of tree branches was assessed by bootstrap analysis with 1,000 replicates.

### Antifungal drug susceptibility testing

The susceptibility of *C*. *lusitaniae* isolates was determined by Etest for amphotericin B, fluconazole, voriconazole, and caspofungin according to the manufacturer's instructions (bioMérieux, France) and as described previously [24]. Bloodstream isolates were also tested for micafungin and anidulafungin, by Etest and for caspofungin and micafungin by Vitek2 yeast identification system. Quality control was ensured by testing *C*. *albicans* ATCC90028, *C*. *parapsilosis* ATCC22019 and *C*. *tropicalis* ATCC750 [24]. As yet, there are no interpretive susceptibility breakpoints available for *C*. *lusitaniae*.

### Statistical analysis

Statistical analysis was performed by using Fisher’s exact test or chi-square test as appropriate and probability levels <0.05 by the two-tailed test were considered as significant. Statistical analyses were performed by using WinPepi software ver. 11.65 (PEPI for Windows, Microsoft Inc., Redmond, WA, USA).

## Results

### Prevalence and phenotypic and molecular identification

Of 7068 yeast isolates tested during the 7-year study period, 134 isolates were identified as *C*. *lusitaniae* (Table 1). The occurrence of *C*. *lusitaniae* isolates varied from 1% in 2012 to 2.9% in 2015 while the occurrence of blood stream isolates varied from 0% in 2012 to 0.6% in 2011 and 2014 (Table 1). The overall prevalence of *C*. *lusitaniae* among yeast species isolates was 1.89%. The largest number of *C*. *lusitaniae* isolates were obtained from sputum samples (n = 54) followed by urine (n = 29) and bloodstream (n = 25). The remaining 26 *C*. *lusitaniae* isolates were obtained from wound/rectal/ear swabs (n = 11), bronchoalveolar lavage (BAL) (n = 4), peritoneal fluid (n = 3), nasopharynx swab (n = 2) and other specimens (n = 6). A total of 990 bloodstream isolates were recovered from 990 candidemia patients during the study period. Thus, the prevalence of *C*. *lusitaniae* among blood stream isolates was higher (25 of 990, 2.5%) than other specimen types (109 of 6078, 1.8%), however, the difference was not statistically significant (*P* = 0.119). None of the bloodstream isolates came from cancer patients. Of 25 bloodstream *C*. *lusitaniae* isolates, 11 were recovered from neonates with nine isolates originating from one hospital (Hospital A) and two isolates from another hospital (Hospital B). All (n = 134) *C*. *lusitaniae* isolates produced white to cream-colored colonies on Sabouraud dextrose agar at 30°C and shades of pink-colored colonies on CHROMagar Candida (S1 Fig). The wet mount examination showed ovoid to sub-globose budding cells with abundant pseudohyphae. All isolates were identified as *C*. *lusitaniae* by Vitek 2 yeast identification with 97–99% probability. The identity of bloodstream isolates was confirmed by MALDI-TOF MS.

**Table 1:** Distribution of total and bloodstream *C*. *lusitaniae* isolates among clinical yeast isolates screened during January 2011 to December 2017.

Year of	No. of yeast	No. of *C*. *lusitaniae*	No. of bloodstream	No. of *C*. *lusitaniae*
isolation	isolates tested	isolates detected	*C*. *lusitaniae* isolates	isolates from neonates
2011	926	26	6	3
2012	924	9	0	0
2013	1052	18	4	1
2014	869	19	5	3
2015	1068	31	5	2
2016	1196	17	4	2
2017	1033	14	1	0
Total	7068	134	25	11

**Fig 1:**
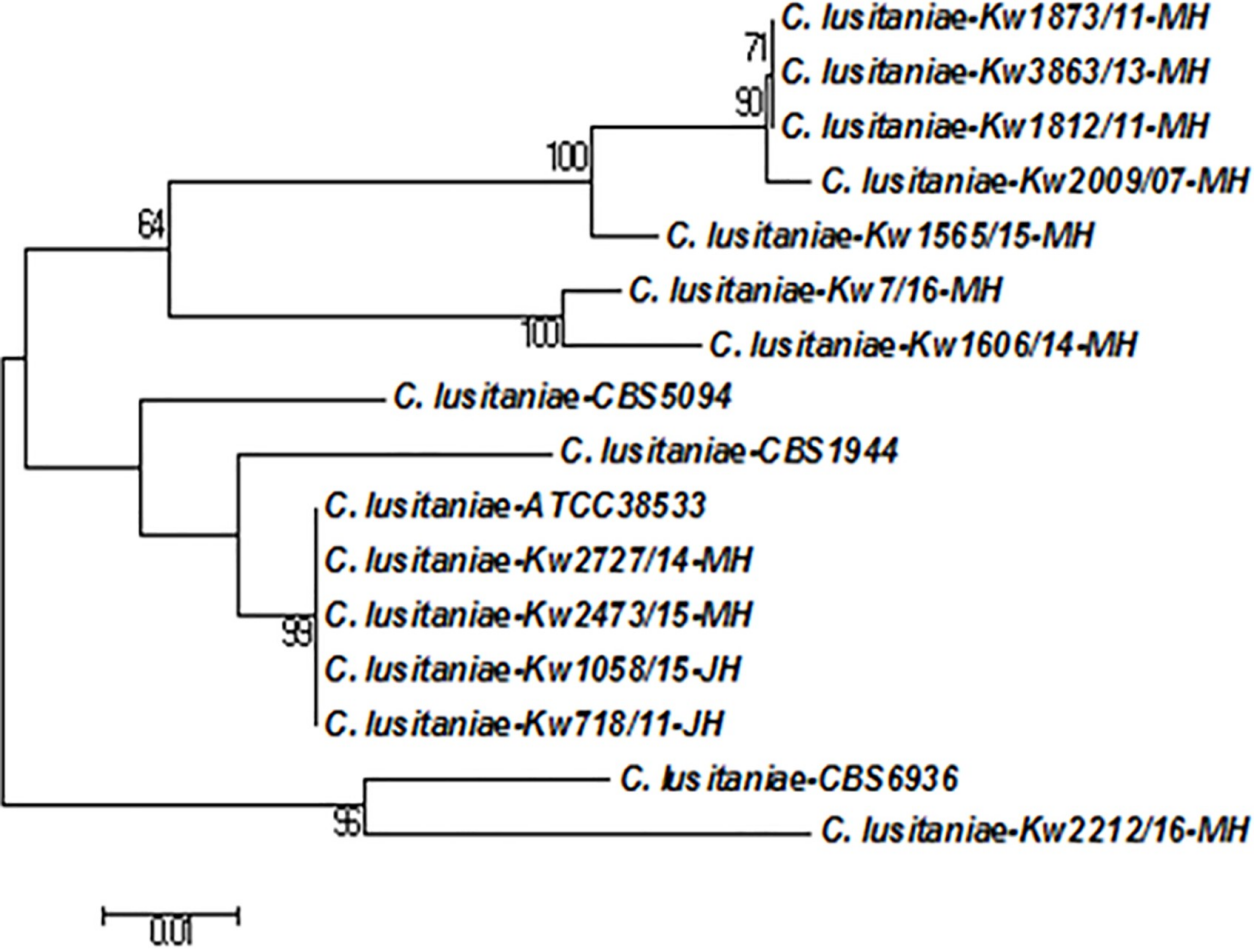
Neighbor-joining phylogenetic tree based on ITS region of rDNA sequence data for clinical *C*. *lusitaniae* strains isolated from nine neonates from Hospital A in Kuwait together with four reference strains. The sequence data for *C*. *lusitaniae* strains isolated from 2 neonates from Hospital B during the study period and one archived *C*. *lusitaniae* strain isolated previously from a neonate from Hospital A were also used for comparison purpose. The numbers on the node branches are bootstrap frequencies.

The PCR amplification performed with CLUSITF and CLUSITR primers yielded an expected size amplicon of nearly 242 bp with DNA extracted from two reference strains (CBS 1944 and CBS 4413) of *C*. *lusitaniae* only while no amplicon was obtained with genomic DNA prepared from reference strains or well characterized clinical isolates of *C*. *dubliniensis*, *C*. *albicans*, *C*. *parapsilosis*, *C*. *orthopsilosis*, *C*. *glabrata*, *C*. *krusei*, *C*. *tropicalis*, C. *guilliermondii*, *C*. *kefyr*, *C*. *haemulonii*, *C*. *duobushaemulonii*, and *C*. *auris*, as expected (S2 Fig). Similarly, no amplicon was also obtained with DNA from *C*. *africana*, *C*. *metapsilosis*, *C*. *conglobata*, *C*. *utilis* and with human DNA. The ITS region of rDNA sequences from 11 bloodstream isolates from neonates (Gen Bank accession numbers LS999909 to LS999920) exhibited maximum identity with corresponding sequences from reference strains of *C*. *lusitaniae* and not with other *Candida* species, as expected. The sequence comparisons also indicated inter-strain variations among clinical *C*. *lusitaniae* isolates from Kuwait. This prompted us to perform molecular fingerprinting studies by comparing ITS region of rDNA sequences. The data showed that 11 isolates exhibited seven different sequence types indicating that many isolates were clonally unrelated (Fig 1).

### *C*. *lusitaniae* candidemia in neonates

During the study period, 11 cases of neonatal candidemia due to *C*. *lusitaniae* were identified. Nine of them occurred in the Neonatal Intensive Care Unit (NICU) of Hospital A and are described in detail here. The salient clinical findings and antifungal susceptibility data for eight patients (clinical details for one patient were not completely available) are presented in Table 2. Seven of eight neonates (including twins, Case 1 and Case 2) were preterm with gestational age varying between 24 to 36 weeks. The birth weight ranged between 600 to 2850 g. All neonates received prior antibiotics (ampicillin and amikacin) and had at least one catheter in place. With one exception (Case 1), all neonates developed bacterial septicemia prior to developing candidemia and received multiple antibiotics for varying periods. The intervening period between date of birth and diagnosis of candidemia ranged between 10 to 60 days (geometric mean ± standard deviation, 27.72 ± 16.12 days). Seven neonates had bacteremia 1 to 44 (5.56 ± 15.15) days before developing candidemia. All patients received amphotericin B or its lipid formulation (AmBisome) as primary therapy for varying duration. It was followed by fluconazole in all cases and additionally by caspofungin in two cases. Despite treatment, three of eight (37.5%) neonates died. Case 1 and Case 2 were twins, both developed *C*. *lusitaniae* candidemia and succumbed to infection despite treatment. The DNA isolated from the blood sample of case 2 (yielding *C*. *lusitaniae* Kw1873/11) was also used for PCR-sequencing of ITS-1 region of rDNA, as described previously [23] and the DNA sequence data of the ITS-1 region matched completely with the corresponding sequence from *C*. *lusitaniae* Kw1873/11.

**Table 2:** Salient features of 9 cases of *C*. *lusitaniae* fungemia in neonates and susceptibility profiles of the isolates.

Case	DOB	GA (wks/sex)	Birth weight (g)	Date of bacterial septicaemia	Date of Candidemia	*C*. *lusitaniae* isolate no.	Catheters	Antibacterials used	Antifungals used	Outcome	Method	Minimum Inhibitory concentration (μg/ml) by Etest					
												AP	FL	VO	CS	MYC	AND
1	7/5/11	24/M	600	1/6/11MRSE D-25	17/5/11 D-10	Kw 1812/11	CVC,UAC UVC	AMP+AK,VAN, MEM	Ambisome	Expired 14/8/11 D-89	E-test	0.016	0.5	0.016	0.19	0.047	0.032
											Vitek	≤ 0.25	≤ 1	≤ 0.12	0.5	0.12	NA
2	7/5/11	24/M	625	20/5/11 *S*.*epidermidis* D-13	23/5/11 D-16	Kw 1873/11	CVC, UAC, UVC	AMP+AK,TEC,TZP	Ambisome, FL	Expired 23/6/11 D-31	E-test	0.016	0.5	0.008	0.125	0.094	0.023
											Vitek	≤ 0.25	≤ 1	≤ 0.12	≤ 0.25	0.12	NA
3	20/10/13	28/F	1200	14/11/13 *Enterobacter* D-25	17/11/13 D-28	Kw 3863/13	UVC	AMP+AK,TZP, VAN	Ambisome, FL, Cs	Discharged	E-test	0.002	0.19	0.008	0.094	0.064	0.064
											Vitek	≤ 0.25	≤ 1	≤ 0.12	≤ 0.25	0.12	NA
4	N.A	N.A	N.A	N.A	11/5/14	Kw 1606/14	N.A	N.A	N.A	N.A	E-test	0.023	0.5	0.004	0.094	0.125	0.047
5	12/8/14	32/M	1500	27/8/14 *P*.*aeruginosa* D-15 24/9 *E*.*faecalis*+ *P*.*aeruginosa* D-43	4/9/14 D-23	Kw 2727/14	CVC, Urinary catheter	AMP+AK,MEM	AP, FL, CS	Expired 25/9/14 D-21	E-test	0.016	0.25	0.006	0.25	0.032	0.016
											Vitek	≤ 0.25	≤ 1	≤ 0.12	≤ 0.12	0.12	NA
6	11/3/15	25/F	780	27/3/15 *S*.*epidermidis* D-16, 22/5 *K*.*pneumoniae* D-72	10/5/15 D-60	Kw 1565/15	CVC	AMP+AK MEM,VAN	Ambisome, FL	Discharged	E-test	0.094	0.75	0.002	0.016	0.032	0.032
											Vitek	≤ 0.25	≤ 1	≤ 0.12	≤ 0.25	0.12	NA
7	3/7/15	36/F	2280	10/8/15 *K*.*pneumoniae* D-38	14/8/15 D-42	Kw 2473/15	─	AMP+AK,MEM	AP, FL	Discharged	E-test	0.064	0.75	0.023	0.19	0.125	0.032
											Vitek	≤ 0.25	≤ 1	≤ 0.12	≤ 0.25	0.12	NA
8	23/11/15	41/M	2850	24/12/15 *S*.*epidermidis* D-31	25/12/15 D-32	Kw 7/16	CVC	AMP+AK TZP,MEM	Ambisome, FL	Discharged	E-test	0.023	0.19	0.003	0.19	0.032	0.094
											Vitek	≤ 0.25	≤ 1	≤ 0.12	≤ 0.25	0.12	NA
9	28/5/16	27/F	1030	26/6/16 *K*.*pneumoniae* D-29	9/7/16 D-42	Kw 2212/16	CVC	AMP+AK TZP,MEM	Ambisome, FL	Discharged	E-test	0.032	0.5	0.003	0.012	0.047	0.023
											Vitek	≤ 0.25	≤ 1	≤ 0.12	≤ 0.25	≤ 0.06	NA

Abbreviations:-DOB-date of birth, GA-gestation age, M-male, F-female, SVD-spontaneous vaginal delivery, MRSE-methicillin resistant *Staphylococcus epidermidis*, CVC-central venous catheter, UAC-umbilical arterial catheter, UVC-umbilical venous catheter, AMP-ampicillin, AK-amikacin, VAN-vancomycin, MEM-meropenem, TEC-teicoplanin, TZP-piperacillin tazobactam, AmBisome- ambisome, AP-amphotericin B, FL-fluconazole, CS-caspofungin, NA- Not available in Vitek. 2 AST-YS07; N. A., not available.

### Antifungal susceptibility

Although there are no validated EUCAST or CLSI susceptibility breakpoints for *C*. *lusitaniae*, all bloodstream isolates appeared susceptible to amphotericin B, fluconazole, voriconazole, caspofungin, micafungin and anidulafungin by Etest (Tables 2 and 3). Etest generally yielded lower MICs than Vitek 2, particularly for amphotericin B (Table 2). The geometric mean ± MIC values for amphotericin B, fluconazole, voriconazole (n = 102), and caspofungin were as follows: 0.037 ± 0.072 μg/ml, 0.49 ± 2.32 μg/ml, 0.01 ± 0.09 μg/ml and 0.16 ± 0.08 μg/ml, respectively. Only two *C*. *lusitaniae* isolates exhibited fluconazole MICs of 16 μg /ml and 24 μg /ml (Table 3).

**Table 3:** Antifungal susceptibility profile of clinical *C*. *lusitaniae* isolates.

Antifungals	No. isolates tested	MIC Range (μg/ml)	GM ± SD (μg/ml)
Amphotericin B	129	0.002–0.38	0.037 ± 0.072
Fluconazole	130	0.016–24*	0.49 ± 2.32
Voriconazole	102	0.002–0.5	0.01 ± 0.09
Caspofungin	118	0.008–0.5	0.16 ± 0.08

*2 isolates showed MIC values of 16 and 24 μg/ml.

## Discussion

In this study, we describe the prevalence of *C*. *lusitaniae* in clinical yeast species and its role in neonatal fungemia in Kuwait. For rapid and unambiguous identification, a simple, low-cost (~1 US$ per sample excluding the cost of culture and personnel time) *C*. *lusitaniae*-specific PCR assay was developed which could be completed within 4 hours using basic PCR and gel electrophoresis equipment that are readily available in routine mycology laboratories.

*C*. *lusitaniae* has attracted world-wide attention since some strains isolated previously were found to be resistant to amphotericin B [3, 4, 5, 25]. In the present study, *C*. *lusitaniae* was isolated from diverse clinical specimens suggesting its ability to colonize different anatomical sites, and thus underscoring its potential role as a nosocomial pathogen [26, 27]. Similar to other *Candida* species, *C*. *lusitaniae* also enters the host via the gastrointestinal, genitourinary and respiratory tracts or through intravascular catheters and the associated risk factors for invasive infections are not very different [28, 29]. Considering the fact that *C*. *lusitaniae* forms only a minor component of human yeast flora (Table 1), its isolation from sterile sites assumes a greater clinical significance as compared to *C*. *albicans* or some other commonly encountered species. The low level of prevalence of *C*. *lusitaniae* is also reflected by the fewer number of cases of invasive infections caused by this species in the hospitalized patients [30–36]. In a comprehensive laboratory-based multicenter surveillance study determining prevalence of *C*. *albicans* and non-*albicans Candida* species among bloodstream isolates, the rate of occurrence of *C*. *lusitaniae* was <2% [33]. Pfaller et al [29] recently reported epidemiological data obtained from Prospective Antifungal Therapy (PATH) Registry for invasive candidiasis caused by non-*albicans Candida* species in North America. *C*. *lusitaniae* was associated with 1.6% (n = 41) of 2496 invasive infections.

*C*. *lusitaniae* is also an uncommon cause of fungemia in neonates with few exceptions. A review of 59 fungemic neonates during 1994–2000 in Greece showed that only two (3.3%) cases were caused by *C*. *lusitaniae* [37]. In a comprehensive prospective observational study that included infants of ≤1000g birth weight, 137 infants developed candidiasis, and in only one (0.7%) case infection was caused by *C*. *lusitaniae* [38]. Like-wise, in an international, prospective study on epidemiology of invasive candidiasis, *C*. *lusitaniae* was isolated from 4% (n = 196) of the pediatric patients but not from the any of the 25 neonates [39]. On the contrary, *C*. *lusitaniae* was found to be more frequent among 45 patients of <1 year of age causing 13.3% (n = 6) of invasive infections in PATH registry data [29]. In a retrospective analysis of 318 bloodstream yeast infections among neonates in Kuwait during 2011–2017, 11 (3.45%) were caused by *C*. *lusitaniae* including 9 described here. Other studies from the Middle East Region have also reported invasive *C*. *lusitaniae* infections. Taj-Aldeen et al. [40] reported two cases of *C*. *lusitaniae* among 17 pediatric patients from Qatar. Both the patients recovered following therapy with lipid formulation of amphotericin B and/or fluconazole (Cases 13 and 14, Table 4). In another study from Saudi Arabia, *C*. *lusitaniae* was recognized as a cause of invasive candidiasis in 5 (4 in neonates and one in an infant) of 129 pediatric patients analyzed retrospectively [41].

**Table 4:** Summary of cases of *C*. *lusitaniae* fungemia an deep-seated infections reported in literature in neonates and infants.

Case Nos.	Reference	Country	Age, Sex	Source of isolation	Underlying disease or condition	Chemotherapy/ steroids /neutropenia	Intravascular catheter	Prior antibiotics	Antifungal therapy	Outcome
1	Christenson et al.; 1987 [44]	USA	2 m, M	Bloo d, RT	Congenital heart disease	Yes	Yes	Yes	AP	Recovery
2	Sanchez & Cooper; 1987 [57]	USA	7 d, M	Blood, Urine, CSF	Prematurity	No	Yes	Yes	AP	Recovery
3	Yinnon et al; 1992 [58]	USA	14 d, M	Blood, Urine, Catheter	Prematurity, CVL	No	Yes	Yes	AP, 5FC, KE	Recovery
4	Oleinik et al; 1993 [45]	USA	8 d, M	blood	Prematurity	-	-	-	AP, FL, 5FC	Recovery
5	Nguyen et al; 1996 [30]	USA	4 m, M	Blood, Catheter	CVL, cardiac surgery	-	Yes	-	AP	Recovery
6	Fowler et al; 1998 [27]	USA	21 d, NA	Urine	Prematurity	No	Catheter	Yes	AP, FL, 5FC	Recovery
7	Fowler et al; 1998 [27]	USA	35 d, NA	blood, Urine, CSF	Prematurity	No	Catheter	Yes	AP, 5FC, FL	Death
8	Fowler et al; 1998 [27]	USA	35 d, NA	blood	Prematurity	No	Yes	Yes	AP, FL, 5FC	Recovery
9	Levy et al; 2002 [59]	USA	3 m, M	Blood, Urine	Chronic granulomatous disease	No	-	Yes	IT, AP, FL	Death
10	Viudes et al; 2002 [31]	Spain	46 d, F	Blood, urine	Prematurity, Congenital nephrotic syndrome	Yes	-	Yes	L-AmB	Recovery
11	Favel et al; 2003 [49]	France	12 d, M	Blood, Urine, Nephrostomy catheter	Prematurity	No	Yes	Yes	L-AmB, AP, FL	Death
12	Estrada et al; 2006 [60]	USA	3 m, M	Lymph node	Chronic granulomatous disease	-	-	Yes	FL	Recovery
13	Taj-Aldeen et al; 2014 [40]	Qatar	7 m, F	Blood	Malabsorption	-	-	-	L-AmB, FL	Recovery
14	Taj-Aldeen et al; 2014 [40]	Qatar	20 d, M	Blood	Motor development delay	-	-	-	L-AmB	Recovery
15	Gautam et al; 2014 [61]	Nepal	7 d twins, F & M	blood	Prematurity	Yes	Yes	Yes	FL	Recovery
16	Chorro-Mari et al; 2015 [43]	UK	189 d	NA	Prematurity, Pyelonephritis				L-AmB, MYC	NA
17	Sariguzel et al; 2017 [62]	Turkey	8 m, M	CSF	Meningitis,Ventricular drainage	Yes	-	Yes	FL	Death

Abbreviations:- M-male, F- female, d- day, w-week, m-month, y- year, NA- not available, CVL- central venous line, ST-solid tumor, VPS- ventriculoperitoneal shunt, RT- respiratory tract, CSF- cerebrospinal fluid, NA- not available,DT-digestive tract AP- amphotericine B, FL- fluconazole, 5FC- 5 fluorocytosine, L-AmB- liposomal Amphotericin B, IT- itraconazole, VO- voriconazole, CS- caspofungin, MYC-micafungin

While echinocandins are the first-line drugs for the treatment of candidemia in adult patients, amphotericin B or its lipid formulations are the recommended options for neonates [42]. In the present study, all neonates with *C*. *lusitaniae* fungemia received amphotericin B or its lipid formulation. Interestingly, two of the neonates were twins (Case 1 and Case 2), developing *C*. *lusitaniae* fungemia on day 10 and day 16 and died despite AmBisome therapy for 89 and 31 days, respectively (Table 2). None of the isolates appeared resistant to amphotericin B. Our experience for treating *C*. *lusitaniae* fungemia is limited. Of the two neonates where caspofungin was used as a salvage therapy, one expired apparently due to other complications. The crude mortality of *C*. *lusitaniae* fungemia in our study may be taken as 33.3% as the three expired neonates also had bacterial septicemia. Recently Chorri-Mari & Christyiansen [43] used micafungin (15 mg/kg/day) in a preterm neonate for treating *C*. *lusitaniae* pyelonephritis for 24 days with no adverse effects. This patient was previously given liposomal amphotericin B without any clinical response.

Contrary to some early reports [44, 45], the occurrence of intrinsic or acquired resistance to amphotericin B is not as common as generally perceived [9, 46]. It is also supported by amphotericin B MIC values of 129 *C*. *lusitaniae* obtained in the present study (0.037 ± 0.072 μg/ml) (Table 3). In an early report, Blinkhorn et al. [28] described two adult cases of *C*. *lusitaniae* fungemia caused by amphotericin B-susceptible strains, who responded to amphotericin B therapy and recovered. The authors emphasized that isolates from patients responding poorly to amphotericin B therapy should be tested for susceptibility. The rarity of amphotericin B resistance among *C*. *lusitaniae* isolates was also supported by the study of Diekema et al. [32]. Of 171 *C*. *lusitaniae* isolates collected globally, only 3 (1.75%) isolates showed MIC values of ≥2 μg/ml against amphotericin B [27]. It is possible that despite in vitro susceptibility of *C*. *lusitaniae* isolates to amphotericin B, there are other factors which may impact clinical efficacy. It has been demonstrated by time-kill curve analysis that susceptible *C*. *lusitaniae* strains may be less prone to fungicidal activity of amphotericin B as compared to *C*. *glabrata* or *C*. *albicans* and thus may be less amenable to treatment particularly in neutropenic cancer patients [12].

It has also been suggested that *C*. *lusitaniae* in its haploid state may be prone to developing drug resistance [47]. The molecular mechanisms of amphotericin B resistance are being elucidated in *Candida* species [48]. There are also reports of acquisition of multidrug resistance in *C*. *lusitaniae* in association with phenotypic switching [49–51]. Favel et al. [49] described a case of fatal kidney infection in a preterm neonate due to *C*. *lusitaniae*. The isolates developed multidrug resistance to amphotericin B and fluconazole during therapy, which was accompanied with switching in colonial morphology. Asner et al. [11] recently reported a case of rapid development of multidrug resistance in *C*. *lusitaniae* to amphotericin B, caspofungin, flucytosine and azoles during treatment of persistent candidemia in an immunosuppressed child. The authors hypothesized that selection pressure created due to use of multiple antifungal drugs might have facilitated successive emergence of resistant strains during the course of therapy. *C*. *lusitaniae* is a heterothallic ascomycetous yeast and mating is only possible between two haploid cells of opposite mating types [47]. Little is known about the mating type that is predominantly associated with human infection or prone to developing resistance or undergo phenotypic switching [51].

As stated above, there are no CLSI/EUCAST antifungal susceptibility breakpoints available for this species. However, several studies have determined epidemiologic cut-off values for *C*. *lusitaniae* to discriminate wild-type and non-wild-type strains [52–54]. Our *C*. *lusitaniae* isolates appeared susceptible to amphotericin B, voriconazole, caspofungin and resistance to fluconazole (≥8 μg/ml) was observed in only two strains (Table 3). Pfaller et al. [55] determined caspofungin susceptibility of 105 *C*. *lusitaniae* isolates obtained over a 4-year period (2001 and 2004) from 91 institutions under global surveillance program. All the isolates were inhibited at a concentration of ≤4 μg/ml. None of our isolates showed MIC value for caspofungin above >0.5 μg/ml (Table 3). We have no comparative susceptibility data for micafungin and anidulafungin, which is a limitation of our study. Here attention may be drawn to a recent study demonstrating loss of fungicidal or fungistatic activity of micafungin in the presence of serum proteins, which is not predicted by MICs in case of *C*. *lusitaniae* [56]. The authors suggested that micafungin and probably other echinocandins should be used with caution against rare *Candida* species including *C*. *lusitaniae*.

A PubMed-based literature search revealed 17 case reports of *C*. *lusitaniae* invasive infections in neonates/children since 1984 (Table 4) [27, 30, 31, 40, 43–45, 49, 57–62]. All had underlying conditions, which included congenital defects (n = 4), prematurity (n = 10) and chronic granulomatous diseases (n = 2) and meningitis/extraventricular shunt (n = 1). Their age ranged between 7 days to 8 months. All patients received antifungal therapy, mainly with amphotericin B (n = 10) or liposomal amphotericin B (n = 5). Three patients were treated with fluconazole alone and one died. Mortality in these 17 cases was about 24% (Table 4).

In conclusion, we have described prevalence and antifungal susceptibility of *C*. *lusitaniae* isolates obtained from diverse clinical specimens over a seven-year period in Kuwait. Additionally, 9 cases of *C*. *lusitaniae* fungemia in neonates are also described. All isolates appeared susceptible to amphotericin B.

## Supporting information

S1 Fig **Pink-colored colonies of five *Candida* species on CHROMagar Candida:** (a) *C*. *auris*, (b) *C*. *famata*, (c) *C*. *guilliermondii*, (d) *C*. *lusitaniae*, strain No. Kw 1812/11-MH, (e) *C*. *lusitaniae* strain No. Kw 2212/16-MH and (f) *C*. *glabrata*.
(DOCX)

S2 Fig Agarose gel of PCR amplicons obtained with *C. lusitaniae*-specific (CLUSITF and CLUSITR) primers and genomic DNA from reference strains of *C. dubliniensis* (lane 1), *C. albicans* (lane 2), *C. parapsilosis* (lane 3), *C. orthopsilosis* (lane 4), *C. glabrata* (lane 5), *C. krusei* (lane 6), *C. tropicalis* (lane 7), *C. guilliermondii* (lane 8), *C. kefyr* (lane 9), *C. haemulonii* (lane 10), *C. duobushaemulonii* (lane 11), *C. auris* (lane 12), *C. lusitaniae* CBS 4413 (lane 13)) and *C. lusitaniae* CBS 1944 (lane 14). Lane M is 100 bp DNA ladder and the positions of migration of 100 bp, 300 bp and 600 bp fragments are marked.
(DOCX)
