# Supplementary material for: Candida lusitaniae in Kuwait: Prevalence, antifungal susceptibility and role in neonatal fungemia
Source: PLoS One. 2019 Mar 7;14(3):e0213532. doi: 10.1371/journal.pone.0213532 (PMC6405135; doi:10.1371/journal.pone.0213532)

**S1 Fig.** Pink-colored colonies of five *Candida* species on CHROMagar Candida: (a) *C. auris*, (b) *C. famata*, (c) *C. guilliermondii*, (d) *C. lusitaniae*, strain No. Kw 1812/11-MH, (e) *C. lusitaniae* strain No. Kw 2212/16-MH and (f) *C. glabrata.*


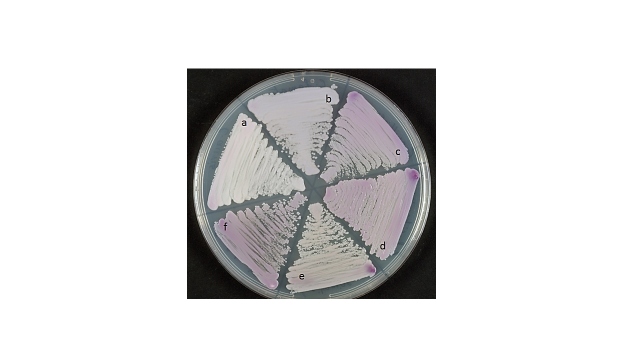

Supplement: S1 Fig — Pink-colored colonies of five Candida species on CHROMagar Candida: (a) C. auris, (b) C. famata, (c) C. guilliermondii, (d) C. lusitaniae, strain No. Kw 1812/11-MH, (e) C. lusitaniae strain No. Kw 2212/16-MH and (f) C. glabrata. (DOCX) [file pone.0213532.s001.docx]
