# Supplementary material for: Candida lusitaniae in Kuwait: Prevalence, antifungal susceptibility and role in neonatal fungemia
Source: PLoS One. 2019 Mar 7;14(3):e0213532. doi: 10.1371/journal.pone.0213532 (PMC6405135; doi:10.1371/journal.pone.0213532)

**Legend**

**S2 Fig. Agarose gel of PCR amplicons obtained with *C. lusitaniae*-specific (CLUSITF and CLUSITR) primers and genomic DNA from reference strains of *C. dubliniensis* (lane 1), *C. albicans* (lane 2), *C. parapsilosis* (lane 3), *C. orthopsilosis* (lane 4), *C. glabrata* (lane 5)*, C. krusei* (lane 6), *C. tropicalis* (lane 7), *C. guilliermondii* (lane 8), *C. kefyr* (lane 9), *C. haemulonii* (lane 10), *C. duobushaemulonii* (lane 11), *C. auris* (lane 12), *C. lusitaniae* CBS 4413 (lane 13)) and *C. lusitaniae* CBS 1944 (lane 14).** Lane M is 100 bp DNA ladder and the positions of migration of 100 bp, 300 bp and 600 bp fragments are marked.


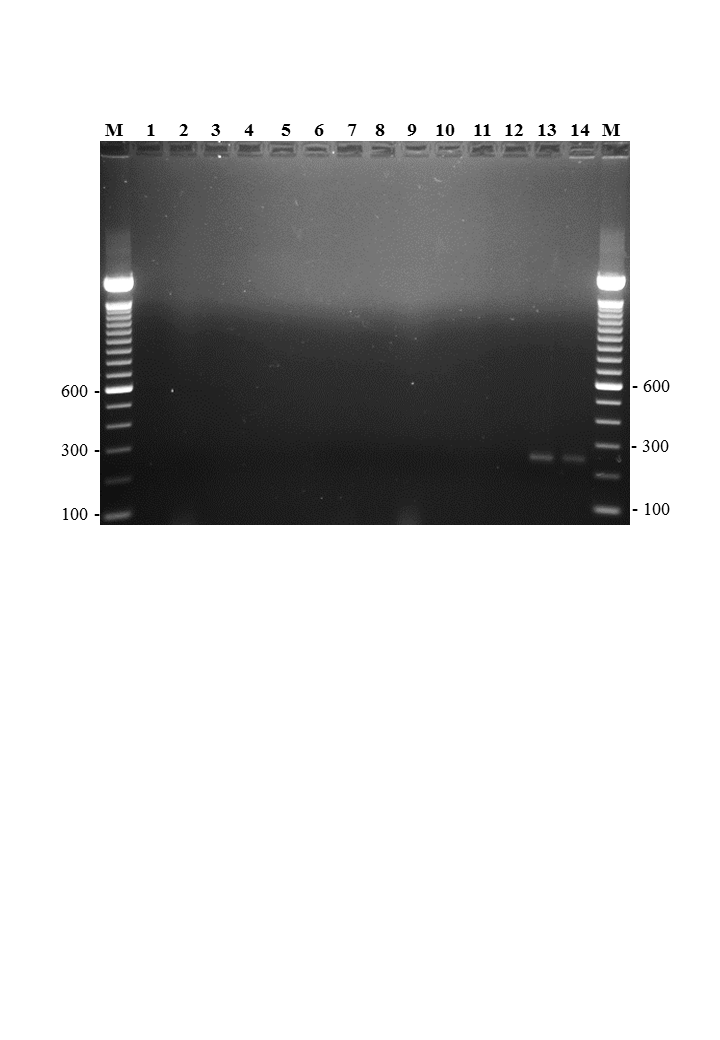

Supplement: S2 Fig — Lane M is 100 bp DNA ladder and the positions of migration of 100 bp, 300 bp and 600 bp fragments are marked. (DOCX) [file pone.0213532.s002.docx]
